# Breast metastasis from nasopharyngeal carcinoma: A case report and review of the literature

**DOI:** 10.3892/ol.2013.1303

**Published:** 2013-04-12

**Authors:** BEN I. LEACH, BONNIE SUN, LYDIA PETROVIC, STEPHEN V. LIU

**Affiliations:** 1 Departments of Medicine, Los Angeles, CA 90033, USA; 2 Surgery, Los Angeles, CA 90033, USA; 3 Pathology, Los Angeles, CA 90033, USA; 4 Division of Medical Oncology, Norris Comprehensive Cancer Center, Keck University of Southern California School of Medicine, Los Angeles, CA 90033, USA

**Keywords:** breast, breast metastasis, Epstein-Barr virus, nasopharyngeal carcinoma

## Abstract

Although often localized at diagnosis, nasopharyngeal carcinoma (NPC) has an established potential for distant metastasis. Breast metastasis from NPC is an uncommon presentation. In the present case study, the fifth reported case of breast metastasis from NPC is presented and the use of Epstein-Barr virus testing is demonstrated for the confirmation of this diagnosis. A 49-year-old female was diagnosed with advanced NPC and developed a unilateral breast mass. The biopsy was indicative of a primary breast carcinoma. Subsequent Epstein-Barr virus testing was positive in the primary tumor and the breast mass, establishing the true diagnosis of NPC metastasis to the breast. In summary, breast metastasis from NPC is an uncommon presentation and Epstein-Barr virus testing is suitable for confirmation of the diagnosis and exclusion of primary breast cancer.

## Introduction

Non-keratinizing, undifferentiated nasopharyngeal carcinoma (NPC; WHO type III) is the most common subtype of NPC and is particularly common in Asia (1). The Epstein-Barr virus has been implicated in the pathogenesis of this subtype and while its presence does not currently guide standard therapy, it may assist in establishing a correct diagnosis (2,3). The majority of cases present with localized disease and although local therapy may be curative, the potential for developing distant metastases is high. The most commonly described sites of metastasis are the lymph nodes, bones, lungs and liver (4). The breast is an uncommon site of metastasis in NPC and represents a diagnostic challenge due to the radiographical similarities to primary breast cancer, a considerably more common cancer. There have been four previously reported cases of NPC with metastasis to the breast (5–7). The present case study reports a fifth case and describes the Epstein-Barr virus testing procedure used to confirm the diagnosis. Informed consent was obatined from the patient.

## Case report

### Clinical presentation

A 49-year-old Vietnamese female was diagnosed with a stage III, WHO type III, NPC upon presentation with diffuse headaches. The patient was treated with curative intent, receiving 6 weeks of definitive radiation and concurrent bolus cisplatin at a dose of 100 mg/m^2^ for 3 doses followed by 3 cycles of adjuvant cisplatin 100 mg/m^2^ and 5-fluorouracil 1000 mg/m^2^/day for 4 days. The patient exhibited a complete response and was disease-free for 18 months, at which point lower back pain developed. Radiographical assessment identified diffuse blastic bone lesions and a biopsy confirmed relapsed metastatic NPC.

### Treatment and clinical course

Following the administration of palliative radiation to a painful sacral lesion, the patient began systemic therapy with weekly paclitaxel 80 mg/m^2^ and cetuximab 250 mg/m^2^, but experienced progression after 6 months. Following this, second line gemcitabine was administered at a dose of 1000 mg/m^2^ on days 1, 8 and 15 in a 4-week cycle and disease control was maintained for 8 months. The patient initially received zoledronic acid with chemotherapy, however, this was later changed to denosumab. Subsequent radiographical procedures then identified progression of the disease with the development of new axillary and iliac adenopathy. A third line therapy using weekly methotrexate (1 mg/kg) was administered; however, progression occurred at 3 months. The patient was then treated in a phase I clinical trial at our institution with the MEK inhibitor GDC-0623 designed to determine the maximal tolerated dose; the disease was controlled for 4 months. Following this, the patient developed a new, palpable, painless mass in the left breast.

### Diagnosis of cancer

**Figure 1:**
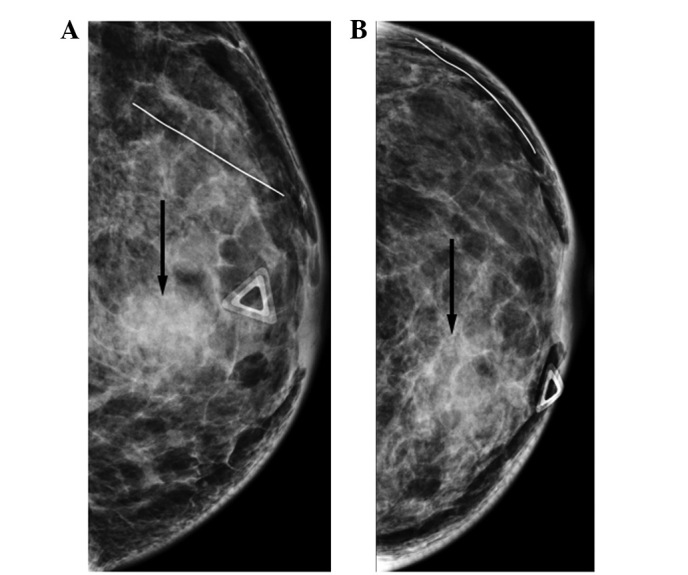
Mammogram revealing the left breast mass. A 5×4.4×1.9-cm irregular hypoechoic mass was observed at the 10 o’clock position (arrow) in the (A) mediolateral oblique and (B) left craniocaudal views.

**Figure 2:**
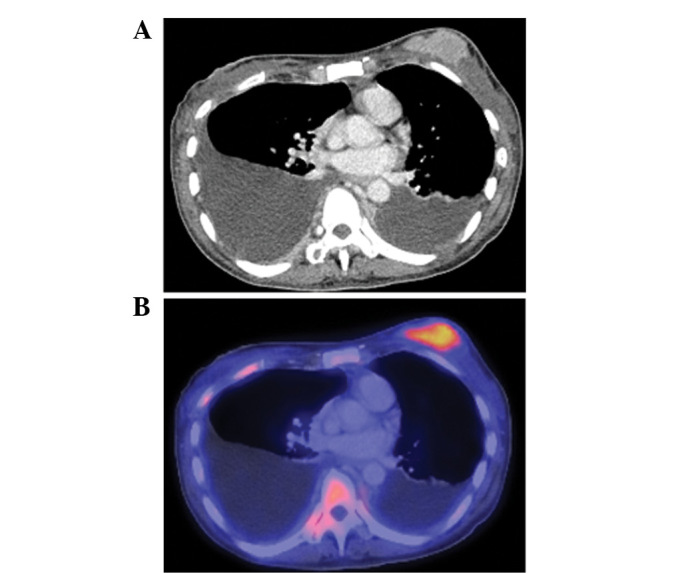
PET-CT of the left breast mass. (A) CT image of the superomedial left breast mass. Other sites of disease were unchanged (data not shown) including the pleural effusion. (B) PET images reveal the left breast mass is hypermetabolic with an SUV of 5.7. PET-CT, positron emission tomography-computed tomography.

Mammography identified a 5×4.4×1.9-cm irregular mass at the 10 o’clock position of the left breast, BI-RADS category 5 (Fig. 1). Positron emission tomography-computed tomography (PET-CT) imaging also identified the new left breast mass (Fig. 2), which was radiographically suggestive of a primary breast carcinoma. The other sites of NPC were unchanged from the prior examinations. An ultrasound-guided biopsy was performed and the analysis revealed malignant cells consistent with a primary breast cancer. Immunohistochemistry revealed no estrogen or progesterone receptor expression and there was no amplification of HER2 expression. The patient received a presumptive diagnosis of a concurrent and separate primary breast carcinoma.

### Histological analysis

**Figure 3:**
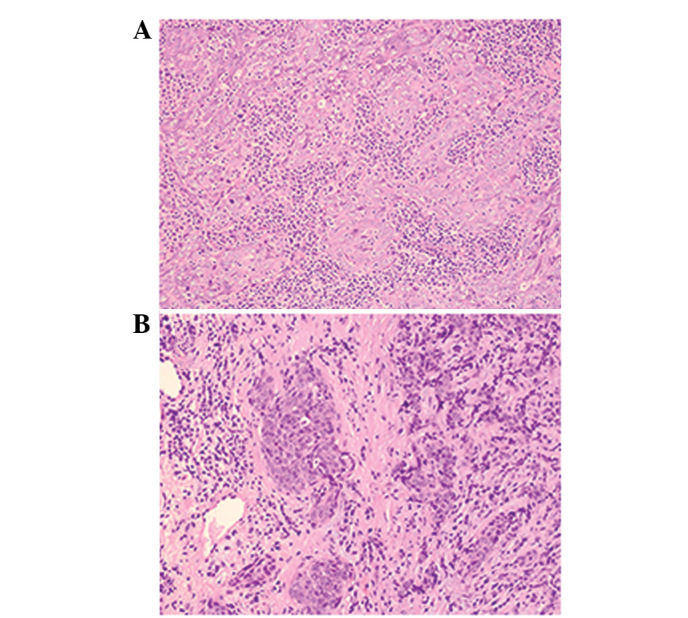
Biopsies from primary and metastatic lesions. (A) Specimen from the primary nasopharyngeal tumor obtained at diagnosis revealing poorly-differentiated malignant (non-keratinizing) cells consistent with nasopharyngeal carcinoma. (B) Specimen from the left breast mass revealing poorly-differentiated malignant cells with similar morphological features to the primary tumor. Subsequent Epstein-Barr virus testing from each specimen was positive. Hematoxylin and eosin (magnification, ×200).

All biopsy specimens were obtained for direct comparison. Following close review, all samples exhibited a similar histological appearance (Fig. 3). Epstein-Barr virus testing was performed by *in situ* hybridization. Slides were incubated, deparaffinized, blocked with 3% hydrogen peroxide, digested, dehydrated and incubated in a prehybridization solution. Slides were then incubated with ribo-probes for EBER1 (8). The primary tumor and the breast mass were Epstein-Barr virus-positive. The diagnosis was changed to progressive NPC metastatic to the breast and the treatment was terminated. At the time of this report, the patient was doing well and beginning fifth line chemotherapy.

## Discussion

Metastasis to the breast from an extra-mammary primary tumor is uncommon, accounting for <2% of tumors identified in the breast (7,9). This holds true for NPC. While distant metastases from NPC are common, only 4 cases of metastasis to the breast have been described (Table I). The potential for misdiagnosis and confusion is high, as primary breast cancer is far more common than NPC and the radiographical appearance of these lesions is often extremely similar (10). Although a biopsy is used to establish the diagnosis, nasopharyngeal biopsies often provide scant tissue for comparison and these two epithelial tumors share a number of histological characteristics. As the treatment for these two types of cancer is vastly different, it is critically important to establish the correct diagnosis.

Epstein-Barr virus is markedly associated with the development of WHO type III NPC and a number of diagnostic modalities have been developed to facilitate the detection of the virus. These include *in situ* hybridization and polymerase chain reaction (11,12). The presence of the Epstein-Barr virus is not necessary to establish a diagnosis of NPC, however, it may be extremely useful for cases where the diagnosis is unclear. In the present case, the detection of the Epstein-Barr virus in the breast mass confirmed the diagnosis of metastatic NPC and facilitated the correct treatment decisions.

Only four cases of breast metastasis from NPC have been previously reported. All four cases described a solitary breast mass that developed following a diagnosis of metastatic NPC. The first patient received an initial radiation treatment to the primary NPC, then developed bone and lung metastases (5). The patient received cyclophosphamide as a salvage therapy, but developed a breast mass with axillary lymphadenopathy. The biopsy was consistent with NPC, and in light of progression, the treatment was changed to cisplatin plus 5-fluorouracil. The patient succumbed to cancer several months later. The second patient also received initial radiation treatment to the nasopharynx and then developed lung metastases (5). Salvage therapy with mitoxantrone was initiated when the patient developed a breast mass with axillary adenopathy. Again, the histological examination was indicative of NPC and the treatment was changed to cisplatin plus 5-fluorouracil. The third patient was treated with systemic chemotherapy for the initial diagnosis of NPC. The individual then presented with bilateral breast masses and pathological lymphadenopathy in the supraclavicular and axillary stations three and a half years later. A diagnosis of metastatic disease was confirmed via biopsy and negative staining for the estrogen and progesterone receptors. In addition, *in situ* hybridization using an Epstein-Barr virus encoded RNA probe was markedly and diffusely positive (6). The fourth patient developed a breast metastasis following the initial diagnosis of NPC, but no details of the treatment were reported (7).

These cases stress the importance of the clinical correlation with biopsy specimens. In the case presented, the breast mass that emerged during therapy was the only sign of disease progression and the confirmatory Epstein-Barr virus testing helped to confirm treatment failure. The radiographical and histological characteristics of breast metastasis from NPC are extremely similar to those of primary breast carcinoma. The clinical context of concurrent or remote NPC may guide the pathologist to consider the diagnosis of this rare diagnosis. In cases of WHO type III NPC, positive Epstein-Barr virus testing provides confirmatory evidence for the diagnosis and direct treatment strategies. As with localized NPC, a multidisciplinary approach is always beneficial to the patient.

## Figures and Tables

**Table I:** Summary of cases of NPC metastasis to the breast.

First author (Ref.)	Year	Age (years)	Initial therapy	Time to relapse (months)	Time to breast metastasis (months)	Survival following diagnosis (months)
Sham *et al*(5)	1986	39	Radiation	17	30	33
Sham *et al*(5)	1987	51	Radiation	27	30	N/R
Driss *et al*(6)	1999	25	Chemotherapy	42	42	N/R
Yeh *et al*(7)	2004	46	Chemotherapy + radiation	N/R	N/R	N/R
Leach *et al*^a^	2009	49	Chemotherapy + radiation	18	39	N/R

a Present case. N/R, not reported; NPC, nasopharangeal carcinoma.
